# The Treatment of Edentulous Jaws to Obtain More Efficient Denture Service1Read before the Illinois State Dental Society, Peoria, Illinois, May 10-12, 1921.

**Published:** 1922-03

**Authors:** G. Walter Dittmar

**Affiliations:** Chicago, Illinois


### THE TREATMENT OF EDENTULOUS JAWS TO OBTAIN MORE EFFICIENT DENTURE SERVICE [1]

BY G. WALTER DITTMAR, D.D.S., CHICAGO, ILLINOIS

I will attempt to discuss but two phases of this subject: (1) surgical treatment, (2) mechanical treatment.

A small percentage of edentulous mouths requires surgical interference to place them in the best condition to wear artificial dentures. Unfavorable muscle attachments may at times be corrected surgically and materially help to make the dentures more efficient. Extremely uneven absorption of the alveolar process often is a decided handicap to both the patient and dental prosthetist. Here again proper surgical interference will be helpful. Bony enlargements (exostosis) of the hard palate or the lingual surface of the mandible may in rare cases need to be removed to obtain a better condition for artificial dentures. Extremely large tuberosities may need to be reduced and other unfavorable defects, deformities or pathological conditions may be treated surgically. Immediately after extracting teeth, cases often present where the smoothing of the crest of the alveolar process—slight reduction of the bony interproximal septi—such as would, if left, be a source of irritation and retard healing and where a number of teeth that approximated each other were re-

---

[1] Read before the Illinois State Dental Society, Peoria, Illinois, May 10-12, 1921.

moved the suturing of the gum flaps is unquestionably good practice, providing there is no serious infection. Occasionally, too, curettage is indicated where a necrotic condition exists or where the recuperative power of the patient is by some systemic derangement unquestionably impaired.

I desire, however, to voice a vigorous protest against an unfortunate and almost criminal practice indulged in by pseudo-oral surgeons and some "exodontists," and the like, viz., the *abuse* of the practice of the so-called "surgical removal of teeth." The irreparable harm that these men do by extensive curettements—the breaking down of the labial and buccal alveolar plates of process, the perforation and infection of the maxillary and other sinuses, the diffusion of bacteria into the system generally, with the final unfortunate dwarfed and contracted base for an artificial plate denture—is a menace so serious that the profession must awaken and "stamp it out."

The leading oral surgeons in our profession—teachers, authors, men in whom we have implicit faith—have been teaching conservatism. They condemn this extreme, insane radicalism. The following is from the President's Address of the Institute of Medicine of Chicago, by Dr. Thomas L. Gilmer.[2]

There has been much unwarranted spectacular operative procedure called "surgery" which removes all adjacent osseous tissues in connection with removal of abscessed and pyorrhea affected teeth. In some cases where there has been extensive bone destruction, operative procedures may be indicated but ordinarily the removal of the tooth suffices. The examination of 2,000 radiographs of edentulous or partially edentulous jaws taken for me in my clinic and private practice in which no curettement or surgical procedures had been observed revealed only three alveolar

---

[2] Also published in *Journal N. D. A.*, VIII (June, 1921), 436.

abscesses which had persisted after the teeth had been removed.

Leading dental prosthetists also protest the dwarfing of the base area for artificial dentures except in those cases where for pathological, aesthetic or mechanical reasons such surgical interference is necessary.

I desire to present a method which was demonstrated to me some months ago by Dr. George E. Everett, of Chicago, which makes it convenient and easy for the prosthetist to keep artificial dentures in a reasonable state of efficiency. This I have termed mechanical treatment. I will confine my remarks primarily to the proposition of rebasing the denture to keep it fitting as well as possible.

The absorption of the alveolar ridge and the changes incident thereto make it necessary to take a new impression whenever the denture becomes loose and troublesome to the patient.

The technic and materials developed during recent years for taking impressions have without question had more to do with the progress made in artificial denture construction than any other of the numerous elements which enter into this important part of our service.

We have now the end results of the work and investigations of such great prosthetic teachers as Bonwill, Walker, Gritman, Snow, Kerr, Christensen, Gysi, the Green brothers, Hoff, Wilson, Prothero, Goslee, Roach, Williams, Hall, Supplee, House, Tench, Monson, and many others. Humanity and our profession owes a great deal to all of these men.

These investigators, authors, and teachers have advocated for the first important step in denture building, viz., taking the impression, such materials as bees' wax, plaster of Paris, modeling compound, com plaster, and the like. All of these properly used have given excellent satisfaction.

To properly carry such materials and confine them in a manner to copy in detail parts necessary, impression trays of many kinds have been devised. The ultimate object sought is a true reverse copy of the tissues when the mouth is closed in normal occlusion, and stress as in biting is applied.

The first important requisite for this technic is an impression tray which practically fits. If the patient has never worn a plate, take an ordinary good plaster impression, make a "cast" and wax base plate; converting the latter into a vulcanite base by using some quick vulcanizable rubber like Dougherty's twenty-minute rubber. This will make an excellent tray. A modeling compound rim can be securely attached for an occlusion rim, or the artificial teeth may be so attached, and with this tray properly trimmed proceed to take the impression. Graft's Base Plate Material nicely fitted to the plaster cast and properly reinforced is a quicker and reasonably good method for making a tray.

The real impression is now to be taken in wax—a special denture impression wax, which Dr. Everett calls a fluid impression compound and which is prepared in three consistencies, soft, medium and hard.

If the patient is wearing a plate or plates that no longer fit properly, such plate or plates should be used as trays for taking the impressions. I will describe the technic for an upper, using the loosely-fitting plate for the impression tray. First scrub the plate with some good kitchen cleanser and a stiff brush. Then swab the inner surface and flanges with chloroform (be certain that it is perfectly clean and dry and slightly warm).

If the base of the denture is not sufficiently large, the flanges not high enough and the distal too short, enlarge by adding Kerr's Perfection Modeling Compound. As this is an important requisite, I will briefly describe the procedure. Use the stick form of Kerr's Compound. Melt the end of the stick in Bunsen flame and fuse to denture base where deficiency exists, to the crest of the flanges and to the distal part of the palatal portion. After adding the compound in this manner immerse the plate in hot water, about 160° F. This will uniformly soften and temper the modeling compound.

Remove plate from hot water and place in the mouth. Have the patient occlude and hold plate firmly in position, then have patient swallow several times and muscle trim by forcibly working the lips and cheek muscles; then muscle trim by having patient open mouth, firmly hold tray in position and have patient forcibly work lips and cheeks. Chill compound and remove plate.

If there is an excess of modeling compound cut it off with a sharp knife. Heat the surface in small Bunsen flame and hot water, as before indicated, and again place in patient's mouth. Have patient again occlude, swallow, and muscle trim.

If there is a deficiency of compound add more and treat in like manner. The object sought is to have a tray that will confine the impression material and force it reasonably close around the periphery, labially, buccally, and distally.

The flanges must not be too high, however, nor the palatal portion too long. The latter must extend slightly onto the soft palate. If the compound is nicely adapted there will be a fair peripheral valve seal and the plate will "stick."

We are then ready to take the wax impression. The wax should be heated in a tin container placed in a double boiler and made fluid. The wax containers are placed in the inner part of the double boiler, the outer part holding water which is heated to the boiling point and kept boiling.

The plate or impression tray is again washed, dried, swabbed with chloroform and warmed to the mouth temperature.

With a long handled "hog hair" brush about three-sixteenths inch in diameter apply the liquid wax to the surface of the tray, painting it on, coating the entire palatal surface and inner surface of the flanges, the crest, the labial and buccal surface of the flanges, and lingual end of palatal portion with the medium wax.

If part of the wax has cooled and has become too stiff, carefully soften the waxed surface over a current of steam coming from the double boiler; then quickly place impression tray in patient's mouth and have him occlude, holding the plate firmly; then have him swallow several times and muscle trim.

After holding the tray for five or ten minutes firmly in position, chill with cold water and carefully remove. It is sometimes hard to remove the impression even at this stage without danger of distorting it. Have the patient assist you, if possible, by forcing air under the impression. Have him hold the lips tightly together and forcibly fill mouth with air so as to fully distend the cheeks. Coughing with the mouth closed sometimes accomplishes the desired effect. If these efforts fail to loosen impression, have patient open mouth, grasp plate in region of first molars with two heavy right-angled hoe excavators and manipulate to loosen the impression, carefully removing it from the mouth.

Wash the impression with cool water and examine. It will most likely show a deficiency of impression material in some places. If a deficiency is detected dry the impression with air and add more of the medium wax in the manner before described. Hold the impression over a current of steam in a manner to slightly soften the entire waxed surface and again carefully insert in patient's mouth; have patient occlude, swallow, and muscle trim, again holding the impression firmly under muscle trim, again holding the impression firmly under biting stress for a few minutes. This will cause the wax to flow and adapt itself to the tissues. Chill again and remove as before described. It will no doubt be more difficult to remove it than previously.

After removal wash again and examine. Dry with cool air and apply a coating of the *softer yellow wax* to the entire surface of the impression. Again carefully project in a cloud of steam and then quickly place in patient's mouth. Follow by the occluding, swallowing and muscle trimming operation and again remove and examine. If a deficiency of impression material exists add more and if there is an excess, cut it off with a hot knife. Place the impression in the mouth and if necessary let the patient wear it for an hour or two, instructing him to occlude on it repeatedly. It is best, however, not to have the patient eat with it because the wax is likely to become softened too much and flow to a degree to spoil the impression, especially if the patient should eat or drink hot food. Just before removing the impression the last time it is a good precaution to have the patient hold cold water in the mouth for a couple of minutes so that the wax will become chilled and hardened. Then test the impression for retentive qualities and if satisfactory, carefully remove as before instructed. Should the plate not "stick" as desired, another application of the softer wax around the periphery will produce a new valve seal and cause the impression to "stick." It is necessary to take the impression under biting stress; therefore, if too many natural occluding teeth are missing, a substantial balanced "bite plate" must be placed on the opposing jaw so that balanced and uniform occluding pressure can be applied.

The next operation is to make a cast, preserving the detail of the crest of the buccal and labial flanges.

After the cast has hardened, smoothe the wax on the labial and buccal portion of the flanges; also on the distal of palatal portion. Invest in vulcanite flask and proceed with the rebasing operation. Relief must of course be provided on the cast where hard areas in the palate indicate.

Wax is an ideal impression material when confined to this technic. The fact that it does not harden so quickly as do the other impression materials and that it flows and equalizes the stress on the tissues when the patient wears the impression denture for an hour or two is a decided advantage.

For lower impressions the technic is practically the same. Prepare the tray or plate so it will have as large a base as is consistent with the conditions and take the impression with wax, using the medium and hard instead of the soft and medium, though at times where the tissues are very soft and flabby or where greater detail is desired the soft wax is indicated.

While this technic may seem like a tedious and time-consuming process, I assure you the results are well worth the effort. The comfort the patient enjoys and the time the dentist saves in making subsequent adjustments, together with the general satisfaction to all concerned, are far more than adequate compensation for the time and effort spent.

Thus instead of resorting to excessive surgery and reducing the alveolar processes to any great extent, cutting away tissue which it would take years to absorb naturally, it is far better to *rebase the dentures frequently,* and for this in particular Dr. Everett's technic and denture impression wax are unusually good.—*Journal N. D. A.*, February, 1922.

